# Alternative Methods of the Largest Lyapunov Exponent Estimation with Applications to the Stability Analyses Based on the Dynamical Maps—Introduction to the Method

**DOI:** 10.3390/ma14237197

**Published:** 2021-11-25

**Authors:** Artur Dabrowski, Tomasz Sagan, Volodymyr Denysenko, Marek Balcerzak, Sandra Zarychta, Andrzej Stefanski

**Affiliations:** Division of Dynamics, Lodz University of Technology, Stefanowskiego 1/15, 90-001 Lodz, Poland; 208516@edu.p.lodz.pl (T.S.); 239742@edu.p.lodz.pl (V.D.); marek.balcerzak.1@p.lodz.pl (M.B.); sandra.zarychta@dokt.p.lodz.pl (S.Z.); andrzej.stefanski@p.lodz.pl (A.S.)

**Keywords:** numerical simulations, stability control, Lyapunov exponents, largest Lyapunov exponent, nonlinear dynamics

## Abstract

Controlling stability of dynamical systems is one of the most important challenges in science and engineering. Hence, there appears to be continuous need to study and develop numerical algorithms of control methods. One of the most frequently applied invariants characterizing systems’ stability are Lyapunov exponents (LE). When information about the stability of a system is demanded, it can be determined based on the value of the largest Lyapunov exponent (LLE). Recently, we have shown that LLE can be estimated from the vector field properties by means of the most basic mathematical operations. The present article introduces new methods of LLE estimation for continuous systems and maps. We have shown that application of our approaches will introduce significant improvement of the efficiency. We have also proved that our approach is simpler and more efficient than commonly applied algorithms. Moreover, as our approach works in the case of dynamical maps, it also enables an easy application of this method in noncontinuous systems. We show comparisons of efficiencies of algorithms based our approach. In the last paragraph, we discuss a possibility of the estimation of LLE from maps and for noncontinuous systems and present results of our initial investigations.

## 1. Introduction

Lyapunov exponents are invariants characterizing numerous aspects of nonlinear systems’ dynamics from complexity, stability, loss of information about a system’s dynamical state, the type and structure of attractor—manifold to which the solution tends. The full spectrum of LE consists of a number of indicators equal to the analyzed system’s dimension. As Lyapunov exponents contain information about the limit of an exponential change of initial perturbation for infinite time range, procedures of LE estimation are very time intensive. Therefore, new methods which could increase the efficiency of LE estimation are still being developed. Even a comparatively minor improvement of a method means huge time savings. As far as investigations into the stability of dynamical systems are concerned, an application of the largest LE is warranted. Since analyzing the stability of dynamical systems is one of the most important challenges in science and engineering, we decided to attempt a development of the LLE estimation method. In the article, we try to demonstrate that our method is both simple and efficient. Additionally, we present the basics for its development, allowing further increase of the efficiency and potential for application for maps and systems with discontinuities.

While the first numerical study of the system behavior using LE dates back to 1964 and the work of Henon and Heiles [1], the LE estimation methods are still being improved [2,3,4,5,6,7,8,9], as LE’s are employed in many different areas of scientific and engineering research, including: mechanical systems [5,10,11,12,13], electrical systems [14,15,16] bioengineering [17], refs. [18,19] astronomy and astrophysics [20,21], materials [22], neuronal models investigations [23,24,25] optimal control [26], time series analyses [27], systems with different types of discontinuities [28,29,30,31], systems with parametric oscillations and fluctuating parameters [32], systems with time delay [12,33,34,35,36,37], systems with hidden attractors [38,39], chaotic fractional order derivative systems [40,41], hybrid-type systems [42,43] and synchronization phenomena analyses [44,45,46,47,48,49,50]. Since LE’s are applied in such a wide spectrum of scientific and engineering research, all the studies regarding properties of LE’s are highly justified.

Recently, we have studied different aspects of the nonlinear systems’ control with the use of different new nonlinear methods. We investigated the stability of continuous systems [51] and systems with discontinuities [9,28], control system’s optimization [52], synchronization phenomena of energy flow [48,53,54] and chaos-based control of energy flow [55,56,57]. We have also investigated efficiency of our novel method of Lyapunov spectrum estimation in [58] and showed that it allows for significant computation time savings.

As far as investigations into the stability of dynamical systems are concerned, application of the largest LE is warranted. Aproximately 60% of scientific research utilizes this simpler and faster indicator. In view of the above, we decided to extend studies of LLE’s properties and present the results of our new investigations.

## 2. The Method

**Figure 1:**
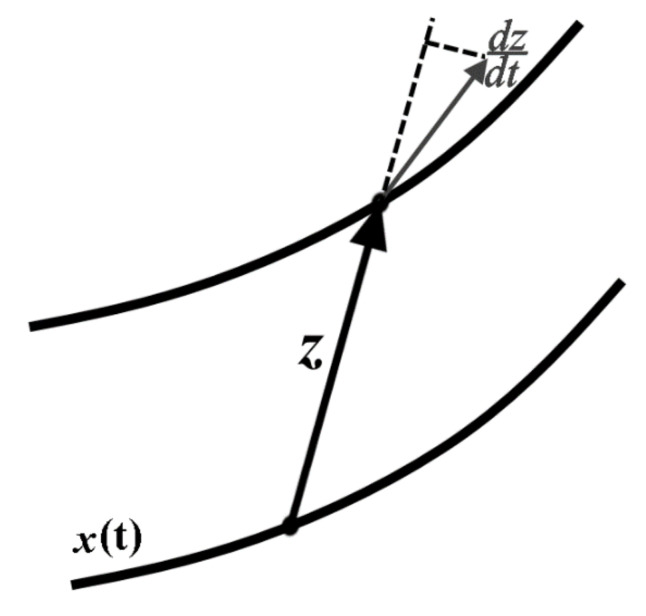
Graphic illustration of the method.

Assume that a dynamical system is described by a set of differential equations in the form:

(1) $$\frac{dx}{dt}=f\left(x,\;t\right)$$

where x is a state vector, t is time and f is a vector field that (in general) depends on x and t. Consider a situation in which the state vector x is disturbed by an infinitesimal perturbation z (Figure 1). Evolution of the perturbation z can be determined by linearization of Equation (1):

(2) $$\frac{dz}{dt}=f\left(x+z,\;t\right)-f\left(x,\;t\right)=\frac{\partial f}{\partial x}\left(x,\;t\right)z=U\left(x,\;t\right)z$$

where $U\left(x,\;t\right)=\frac{\partial f}{\partial x}\left(x,\;t\right)$ is the Jacobi matrix obtained by differentiation of f with respect to x. If the Jacobi matrix was constant, then the evolution of the perturbation z in directions of subsequent eigenvectors would be specified by corresponding eigenvalues of that matrix. However, as long as the system (1) is nonlinear, the Jacobi matrix varies along the trajectory meaning that the evolution of the perturbation z cannot be directly predicted from properties of the Jacobi matrix. In such a case, Lyapunov exponents are applied to describe an average rate of expansion or contraction of a perturbation. Consequently, Lyapunov exponents can be treated as generalization of eigenvalues [59] of the Jacobi matrix. Moreover, according to [59] during an evolution of the system, eigenvectors connected with the largest eigenvalue spans the linear subspace which tends to align with the direction of the perturbation $z\left(t\right)$. As such, all the analyses of LLE can be focused on this direction. Following this eigenvalue idea, during numerical integration for each i - step of n integration steps, Equation (2) in the actual $z_{i}$ direction can be presented in the following scalar form:

(3) $$\frac{dz_{i}}{dt}=\lambda_{i}z_{i}$$

where $\lambda_{i}$ tends to the largest eigenvalue of $U\left(x,\;t\right),$ and its average value is equal to LLE.

Equation (3) can be expressed in the form:

(4) $$\frac{dz_{i}}{z_{i}}=\lambda_{i}dt$$

In the case where the perturbation is normalized before each integration step, $z_{i}=1$:

(5) $$dz_{i}=\lambda_{i}dt$$

For *n* numerical integration steps, from Equation (5), averaged perturbation:

(6) $$\frac{\sum_{i=1}^{n}dz_{i}}{n}=\frac{\sum_{i=1}^{n}\left(\lambda_{i}dt\right)}{n}\equiv \frac{\sum_{i=1}^{n}dz_{i}}{n\cdot dt}=\frac{\sum_{i=1}^{n}\left(\lambda_{i}\right)}{n}=\mathrm{LLE}$$

Finally,

(7) $$\frac{\sum_{1}^{n}dz_{n}}{t}=\mathrm{LLE}$$

From formula (7), one can see that LE can be treated as a dimensionless perturbation change averaged per time unit. It constitutes the basis for the first of the new methods (M1). As perturbation change dz is the scalar obtained from the differences between norms of z before and after each integration step, the method can be applied in the estimation of LLE from any given map. Additionally, it can be also applied for all the systems with any given discontinuities.

Moreover, following Equation (3), when the perturbation is normalized before each integration step:

(8) $$\frac{dz}{dt}=v\left(t\right)=\lambda$$

As the value of λ has to be averaged during evolution of the system to obtain LLE, from Equation (8), one can see that LLE equals the averaged speed $\bar{v}$ of perturbation changes:

(9) $$\bar{v}=\mathrm{LLE}$$

The above constitutes a basis for the second of the new methods (M2).

Incidentally, both of the methods can be treated as identical. They differ only in the way the computed values are averaged during numerical integration. In the first one (Equation (7)), the values of dz are summed up and then averaged by division by time t of calculations. Additionally, regarding the averaged speed (Equation (9)), the actual speed is computed and summed up and the final value of LLE is obtained by division by number n of times of integration. As n·dt=t, both of the methods are equivalent.

## 3. Numerical Simulations

### 3.1. Methodology

All the programs for conducting numerical simulations have been written in C++ by means of the Code: Blocks environment. The Runge–Kutta method of the fourth order (RK4) has been used to solve ordinary differential equations. The integration step has been adjusted for each analyzed system separately, based on its own time scale.

We have studied perturbation change averaged per time unit and averaged speed $\bar{v}$ of perturbation change and compared them with three other methods. All of the considered algorithms of the LLE estimation require integration of the system (1) along with the Equation (2) in order to obtain the state vector $x\left(t\right)$ and the perturbation $z\left(t\right)$ in subsequent moments of time. Depending on the method, the vector $z\left(t\right)$ was either normalized before each integration step or normalized only in the case of excessively high or low values of perturbation length $z\left(t\right)$.

All the programs for estimation of the LLE share the same code for integration of the systems (1), (2). The only difference between these programs is the method of the LLE calculation.

#### 3.1.1. Method 1 (M1)

In the first one, the value given by the Equation (7) is calculated after each integration step. Value dz was obtained from the differences between norms of z before and after each integration step. Vector z was normalized before each integration step. 

#### 3.1.2. Method 2 (M2)

In the second case, the value given by Equation (9) is calculated from projection of the vector $\frac{dz}{dt}$ onto the direction of normalized vector z according to formula:

(10) $$\lambda =\frac{\frac{dz}{dt}\cdot z}{{\left|z\right|}^{2}}=\frac{\frac{dz}{dt}\cdot z}{z\cdot z}\;$$

As vector z was normalized before each integration step,

(11) $$\lambda =\frac{dz}{dt}\cdot z$$

#### 3.1.3. Method 3 (M3)

The third case involves application of the classical method [59] for vector ***z*** normalized in the case of excessively high or low values of perturbation length $z\left(t\right)$:

(12) $$\lambda =\frac{1}{t}\;\ln\frac{z\left(t\right)}{z\left(0\right)}$$

#### 3.1.4. Method 4 (M4)

The fourth case is application of the classical method [59] for vector z normalized before each integration step:

(13) $$\lambda =\frac{1}{t}\;\ln z\left(t\right)$$

#### 3.1.5. Method 5 (M5)

The last case is the application of our effective method presented in [58]. In this case, the value given by Equation (9) is calculated from the projection of the vector $\frac{dz}{dt}$ onto the direction of not normalized vector z according to:

(14) $$\lambda =\frac{\frac{dz}{dt}\cdot z}{z\cdot z}$$

In simulation algorithms, conditions for termination of calculations have to be selected. It seems reasonable to finish the estimation procedure if the obtained value of the LLE stabilizes at some fixed value and does not display any relevant fluctuations. In order to measure stabilization of the LLE value, the authors propose to define a buffer of a fixed size. In this research, the buffer capacity equal to 100 was selected. After each calculation step, the current value of the LLE was saved to the buffer. When the buffer was full, the standard deviation of all the LLE values in the buffer was calculated. If the standard deviation related to actual average LLE was below a specified threshold, the value of the LLE was considered as stable and the calculations could be terminated. Failing that, the buffer was cleared and the procedure repeated. The value of the selected threshold corresponded to the desired accuracy of estimation. Lowering the threshold meant higher accuracy, but, consequently, a longer estimation time. Considering the standard deviation threshold, two methods, with relative and not relative deviation value, can be applied. They differ in accuracy of LLE estimation depending on the dynamical state of the system. In the regions of higher absolute LLE values for high accuracy, it proves advantageous to use nonrelative deviation; in the case of quasiperiodic regions, relative value will produce more accurate results. As insignificant differences in values in the periodic and chaotic regions are not of considerable importance, and conversely, detection of the exact bifurcation point is one of the most important considerations in nonlinear systems investigations, relative deviation was applied in our simulations.

As regards the threshold of excessively high or low values of perturbation’s vector z length, the normalization condition was associated with the product of the first two coordinates of vector z. It allowed for introducing a condition, which does not burden simulation procedures much.

### 3.2. Results of Numerical Simulations

In order to verify the presented methods of the LLE estimation, two typical nonlinear systems have been analyzed. What follows are the results obtained for Duffing and Van der Pol systems with external forcing. Since the details that follow are organized in the same manner, in order to avoid repeating the same description, specification of the graphs is provided only once below.

The first type of the graphs that follow provides the obtained values of the LLE along with computation time lengths for all the investigated methods. Ratios of the program execution times $t_{1},\;\ldots ,\;t_{5}$ for all of the five methods represent the execution time of LLE estimation for the specified bifurcation parameter and method, respectively. In the article, we have associated uniform color schemes and types of curves with respective methods.

Subsequently, efficiency analysis is presented. Special efficiency indicators are introduced. Let $T_{1},\;\ldots ,\;T_{5}$ be sums of $t_{i}$ values, presenting the time measured from the beginning of simulations to the moment a specified bifurcation parameter for each of the five methods has been reached. Let us use these values to introduce efficiency indicators:

(15) $$\eta_{i}=\frac{T_{i}}{T_{3}}\;$$

Relations of $\eta_{i}$, with respect to bifurcation parameter of the investigated algorithms M1, M2, M4, M5 as compared to the classical method M3 are presented on subsequent charts. The efficiency gain of the four investigated methods in comparison to the commonly applied method M3 is appreciable.

In the following charts, dependence of LLE on bifurcation parameter is presented along with focused analyses presenting the accuracy of LE estimation for three different dynamical states: periodic, quasiperiodic and chaotic.

### 3.3. Duffing Oscillator

The Duffing oscillator can be described by the following set of differential equations:

(16) $$\left\{\begin{array}{c} {\dot{x}}_{1}=x_{2}\\ {\dot{x}}_{1}=-\beta x_{2}-\alpha x_{1}^{3}+qcos\left(\omega t\right) \end{array}\right.\;$$

Based on Equation (2), the Jacobi matrix is necessary to observe evolution of a perturbation. For the Duffing oscillator, the Jacobi matrix is defined as follows:

(17) $$U=\left(\begin{array}{cc} 0 & 1\\ -3\alpha x_{1}^{2} & -\beta \end{array}\right)\;$$

**Figure 2:**
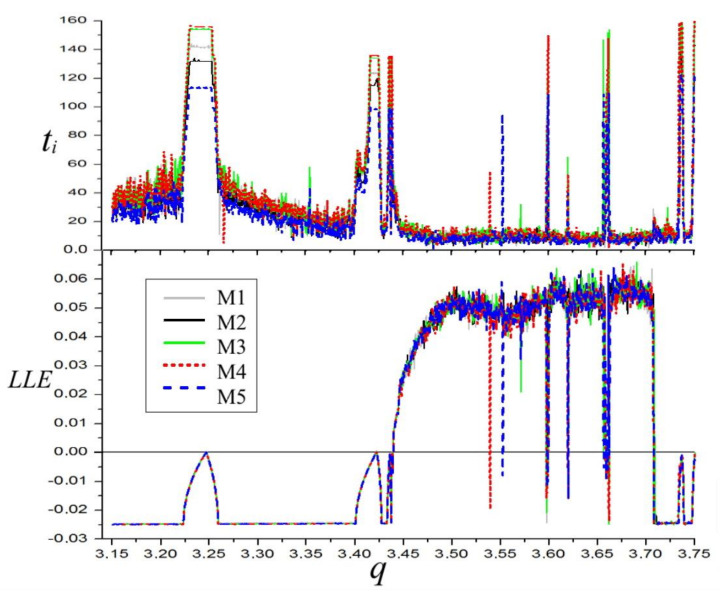
Diagram of the largest Lyapunov exponent of the Duffing system and computation times $t_{i}$[s] graphs. α = 1, β = 0.05, ω = 0.47.

The plot of the LLE for different values of the parameter q and graphs depicting computation time ratios are presented in Figure 2.

It is evident that the longest times occur in chaotic regions, and in instances when the system is approaching bifurcation points. This is related to a longer time which is required to stabilize LLE in a chaotic regime in the first case. In the second case, the main reason was given above and is connected with computing relative or not relative standard deviation in the procedure concluding LLE computations. Since minor differences in values in the periodic and chaotic regions are not highly important, and, conversely, the detection of the exact bifurcation point is one of the most important issues in nonlinear systems investigations, relative deviation was applied in our simulations. Obviously, this increased the time needed to satisfy the required LLE value stability condition.

**Figure 3:**
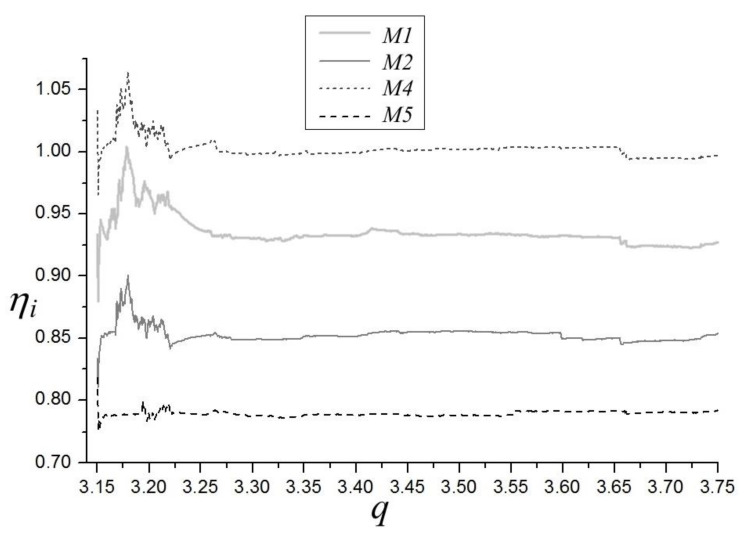
Diagram of efficiencies $\eta_{1},\;\eta_{2},\;\eta_{4},\;\eta_{5}$, of LLE computations of the Duffing system. α = 1, β = 0.05, ω = 0.47.

The efficiency analysis of four methods M1, M2, M4, M5 with respect to M3 is presented in Figure 3. From the method of construction of the $\eta_{i}$ indicators, one can deduce that these values for the specified bifurcation parameter q show the average efficiencies of computations of each method from the beginning of calculations until the parameter q is reached. Therefore, the values $\eta_{i}$ corresponding to the last values of bifurcation parameter present all the average efficiencies of each of the methods. As is evident from Figure 3, only method M4 has efficiency which is not superior to M3. This is to be expected, as M4 is based on M3 and utilizes normalization of perturbation vector in each integration step, while, in M3, the normalization is carried out only in the cases of excessively high or low values of perturbation’s vector z length. The final efficiency $\eta_{4}$ is equal to 0.997. Both of the new presented methods, M1 and M2, offer better efficiency than M3. Method M1, which has the potential to be applied in non-continuous systems, offers the final efficiency $\eta_{1}$ equal to 0.927. Therefore, on average, M1 saves about 7% of the computation time. The effect will be even more pronounced when applied for the maps. Method M2 has the final $\eta_{2}$ equal to 0.853, so it saves on average about 14% of the computation time. Finally, method M5 has the best average efficiency $\eta_{2}$ equal to 0.780, so M5 saves on average about 22% of the computation time. The results for M1, M2, and M5 will be marginally inferior for more complex systems, as shown in [58]. However, they will be invariably superior to M3.

**Figure 4:**
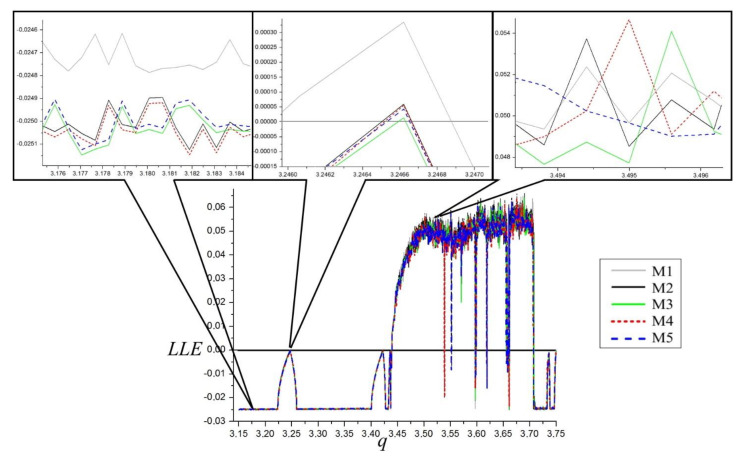
Diagram of accuracy of LLE computations, of the Duffing system. α = 1, β = 0.05, ω = 0.47.

Accuracy comparison of LLE estimation is presented in Figure 3, where the LLE dependence on bifurcation parameter q on a low scale is shown. It can be seen that there exists correspondence between the results of all five of the methods. Higher scale results for the three different dynamical states of the system are presented in the upper part of Figure 4. There is good agreement for M2…M5 methods and only minor differences exist for the M1 method in the periodic and quasiperiodic regions. As these are fourth order level differences, they do not disqualify the M1 method, especially that its efficiency will be considerably higher when applied in LLE estimation from maps. 

### 3.4. The Van der Pol Oscillator

The Van der Pol oscillator can be described by the following set of differential equations:

(18) $$\left\{\begin{array}{c} {\dot{x}}_{1}=x_{2}\\ {\dot{x}}_{2}=\mu \left(-x_{1}{}^{2}+1\right)x_{2}+x_{1}+qcos\left(\omega t\right) \end{array}\right.\;$$

Jacobi matrix was used to simulate evolution of a perturbation according to Equation (2). For the Van der Pol oscillator, the Jacobi matrix is defined as follows:

(19) $$U=\left(\begin{array}{cc} 0 & 1\\ -2\mu x_{1}x_{2}+1 & \mu \left(-x_{1}{}^{2}+1\right) \end{array}\right)\;$$

**Figure 5:**
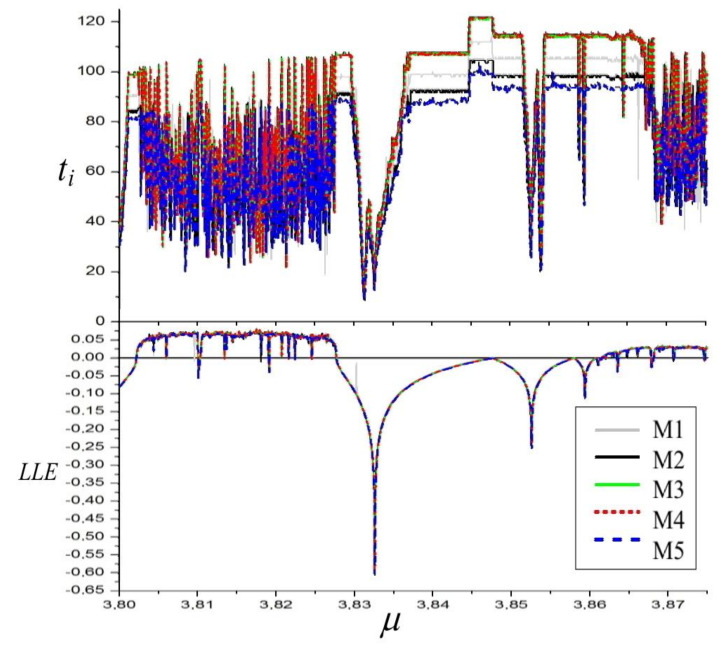
Diagram of the largest Lyapunov exponent of the Van der Pol system and computation times $t_{i}$[s] graphs. q = 12.95, ω = 4.64.

**Figure 6:**
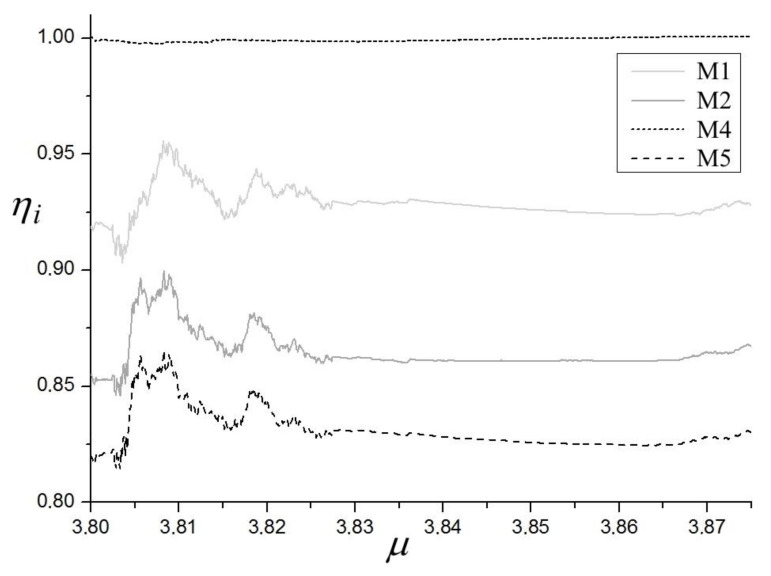
Diagram of efficiencies $\eta_{1},\;\eta_{2},\;\eta_{4},\;\eta_{5}$ of LLE computations of the Van der Pol system. q = 12.95, ω = 4.64.

The plot of the LLE for different values of the parameter μ, together with computation times, is presented in Figure 5, whereas graphs depicting computation time ratios are presented in Figure 6. For the same reasons as in the case of Duffing system, the longest computations appear in chaotic regions and when the system is approaching bifurcation points. These effects connected with the application of the relative deviation can be also observed in the regions with high absolute values of negative LLE. As the demanded accuracy in these regions decreases together with the values of LLE, one can see short computation times in these areas. What is important and also evident from the lower part of Figure 5, the influence of such variable accuracy on the estimated values of LLE is negligible–no significant noise caused by this effect can be observed on the LLE graph.

It can also be seen in Figure 5 that, even in the Van der Pol system, its divergence varies in time of oscillations—as its dumping is nonlinear, less time is needed to stabilize LLE in chaotic and qusiperiodic regions than in the case of the Duffing system. Maximal values of time for Van der Pol are approximately 120 [s], while, for the Duffing system, they are approximately 160 [s]. As divergence of the system is equal to the Lyapunov exponents’ sum, it would appear that the varying divergence could disrupt the stabilization process of LLE values. Apparently, not only does it disrupt the process, but it speeds it up. 

The effects of variable divergence can be also observed in the values of LLE in periodic regions. In the case of the Duffing system, the values of LLE are constant and equal to half of the divergence (the second Lyapunov exponent is equal to LLE). In the case of Van der Pol, there exist no regions of constant LLE.

Efficiency analysis of the four methods M1, M2, M4, M5 with respect to M3 is presented in Figure 6. For the same reasons as in the case of the Duffing system, it is only method M4 that has no superior efficiency compared to M3. Both of the new presented methods, M1 and M2, have better efficiency than M3. Method M1, which has the potential to be applied in non-continuous systems, has the final efficiency $\eta_{1}$ equal to 0.928 (Duffing 0.927). Therefore, on average, M1 saves about 7% of the computation time. The effect will be even more pronounced when applied for maps. Method M2 has the final $\eta_{2}$ equal to 0.867 (Duffing 0.853), so it saves on average about 14% of the computation time. Finally, method M5 has the best average efficiency $\eta_{2}$ Equal to 0.837 (Duffing 0.780), which translates into an average of approximately 16% savings of the computation time. These results confirm conclusions for the Duffing system.

**Figure 7:**
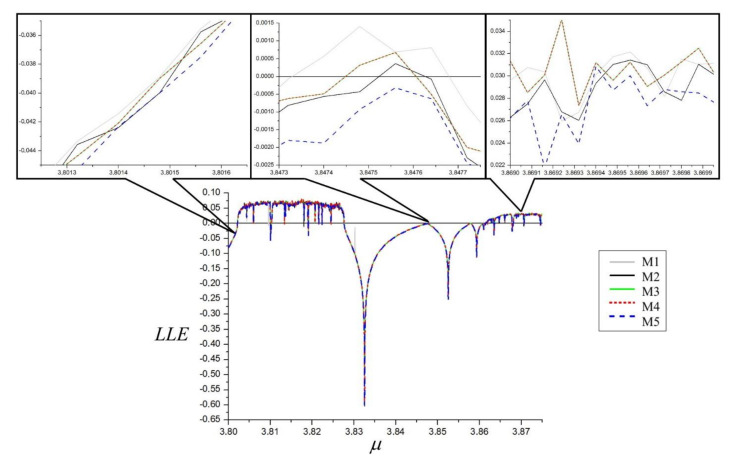
Diagram of the accuracy of LLE computations of the Van der Pol system. q = 12.95, ω = 4.64.

Accuracy comparison of LLE estimation is presented in Figure 7. In the bottom section, one can see LLE dependence on bifurcation parameter q on a low scale. Similarly to the Duffing system, there exists a good agreement between the results of all five methods.

Higher scale results for the three different dynamical states of the system are presented in the upper part of Figure 4. It can be appreciated that, unlike for the Duffing system, the results instead merge and cannot be accurately determined.

## 4. Largest Lyapunov Exponent (LLE) from Maps

As it was proved in [60], with the use of our method, perturbation behavior of a perturbation can be reconstructed based on the time series of the dynamical system, without reconstruction of the Jacobi matrix. It can be combined with the approach presented above and then applied for dynamical maps.

**Figure 8:**
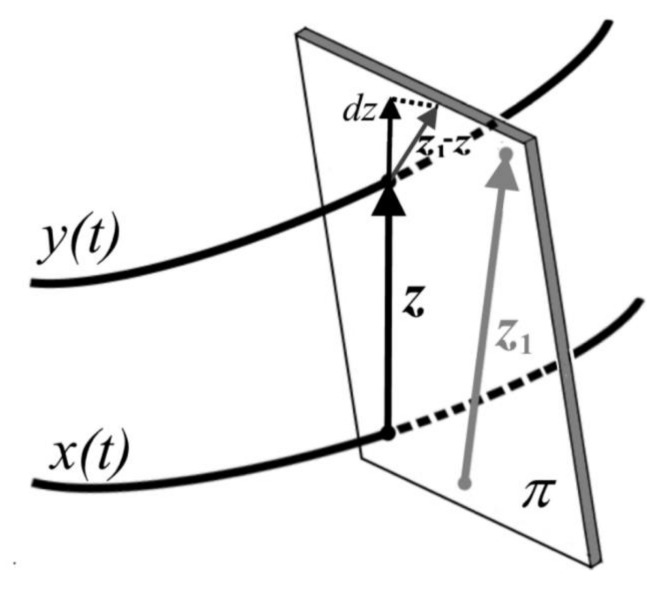
Graphical illustration of the method for maps.

**Figure 9:**
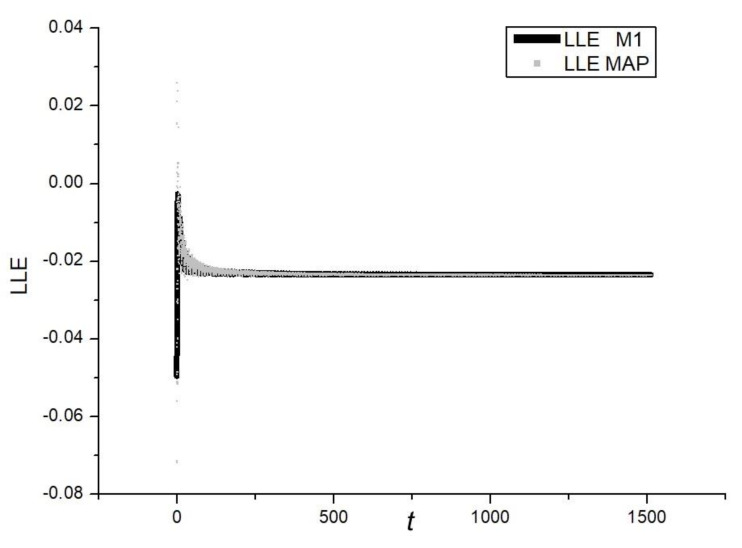
Time series comparison.

The first approach comes directly from application of the method M1. In this case, the value of the sum of perturbations was averaged while the trajectory $x\left(t\right)$ crossed the hyperplane π—see Figure 8. A time series comparison with the method M1 is shown in Figure 9. It can be seen that estimation error is within the same range as for the method M1.

The second approach requires an extended analysis. In Figure 8, a trajectory $x\left(t\right)$ of a dynamical system and the perturbed system trajectory $y\left(t\right)$ can be seen. While these trajectories cross the hyperplane π, one obtains perturbation ***z*** and then next perturbation ***z*_1_** from the next points of crossing trajectories through the hyperplane π. After projection of the difference of the vectors $z_{1}-z$ on to the direction of perturbation z, one obtains a differential dz. It allows for substituting the lengths z and dz into Equation (4) to find λ value. Alternatively, dz can be calculated from the difference of the norms of vectors $\left|z_{1}\right|-\left|z\right|$. However, in this case, the estimation error is expected to be higher. During the evolution of the system, obtained values have to be averaged and then recalculated according to the error correction analysis presented below.

### Error Correction Analysis

Between the trajectory crossing the hyperplane ***π***, there were calculated i steps of numerical integration. During numerical calculation of LLE, in each integration step, values $\lambda_{i}$ are obtained, and then averaged in time in order to obtain LLE. Following reasoning that justified scalar notation of Equation (3), we can continue in the same vein in the case of maps. Then, the value of the proposed indicator for a map is:

(20) $$\lambda_{map}=\frac{z_{i}-z_{0}}{z}\;$$

Consider

(21) $$z_{i+1}=z_{i}+dz_{i}\;and\;dz_{i}=\lambda_{i}z_{i}dt\;$$

(22) $$\frac{\lambda_{map}}{dt}=\lambda_{0}+\lambda_{1}+\ldots +\lambda_{i-1}+\lambda_{i}+\delta \;$$

where *δ* is LLE estimation error.

While the final value of LLE is an average of $\lambda_{i}$:

(23) $$\frac{\lambda_{map}}{idt}=\frac{\lambda_{map}}{T}=\mathrm{LLE}+\frac{\delta }{i}\;$$

where *T* is the time from one to the next crossing the map. To simplify the analysis of the correction error, let us start with *i* = 5. Then,

(24) $$\begin{array}{c} \delta =dt(\lambda_{0}\lambda_{1}+\lambda_{0}\lambda_{2}+\lambda_{0}\lambda_{3}+\lambda_{0}\lambda_{4}+\lambda_{1}\lambda_{2}+\lambda_{1}\lambda_{3}+\lambda_{1}\lambda_{4}+\lambda_{2}\lambda_{3}+\lambda_{2}\lambda_{4}+\lambda_{3}\lambda_{4})+\\ +dt^{2}\left(\lambda_{0}\lambda_{1}\lambda_{2}+\lambda_{0}\lambda_{1}\lambda_{3}+\lambda_{0}\lambda_{1}\lambda_{4}+\lambda_{0}\lambda_{2}\lambda_{3}+\lambda_{0}\lambda_{2}\lambda_{4}+\lambda_{0}\lambda_{3}\lambda_{4}+\lambda_{1}\lambda_{2}\lambda_{3}+\lambda_{1}\lambda_{2}\lambda_{4}+\lambda_{1}\lambda_{3}\lambda_{4}+\lambda_{2}\lambda_{3}\lambda_{4}\right)\\ +dt^{3}\left(\lambda_{0}\lambda_{1}\lambda_{2}\lambda_{3}+\lambda_{0}\lambda_{1}\lambda_{2}\lambda_{4}+\lambda_{0}\lambda_{1}\lambda_{3}\lambda_{4}+\lambda_{0}\lambda_{2}\lambda_{3}\lambda_{4}+\lambda_{1}\lambda_{2}\lambda_{3}\lambda_{4}\right)\;\\ +dt^{4}\lambda_{0}\lambda_{1}\lambda_{2}\lambda_{3}\lambda_{4}\; \end{array}$$

Finally, *n*-th power of *dt* is connected with $\left(\begin{array}{c} i\\ n-1 \end{array}\right)$ combinations of $\mathrm{products}\;\mathrm{of}\;\left(n+1\right)\;\mathrm{of}\;\lambda_{j\;}$, where: j = 0…i−1. Obviously, $\lambda_{j\;}$ are unknown while calculating $\lambda_{map}$. In order to estimate the value of the correction error, we have assumed that $\lambda_{j\;}$ equals the average value $\lambda_{av}$. Then:

(25) $$\delta =\frac{dt\left(\begin{array}{c} i\\ 2 \end{array}\right)\lambda_{av\;}{}^{2}+dt^{2}\left(\begin{array}{c} i\\ 3 \end{array}\right)\lambda_{av\;}{}^{3}+dt^{3}\left(\begin{array}{c} i\\ 4 \end{array}\right)\lambda_{av\;}{}^{4}+dt^{4}\left(\begin{array}{c} i\\ 5 \end{array}\right)\lambda_{av\;}{}^{5}+\ldots +dt^{i-2}\left(\begin{array}{c} i\\ i-1 \end{array}\right)\lambda_{av\;}{}^{i-1}+dt^{i-1}\left(\begin{array}{c} i\\ i \end{array}\right)\lambda_{av\;}{}^{i}}{i}$$

As $\lambda_{av\;}=\mathrm{LLE}$ and for nondimensional *T* = 1 $i=\frac{1}{dt}$, we obtain the final correction error CE:

(26) $$\mathrm{CE}=\overset{i}{\underset{j=2}{\sum }}dt^{j}\left(\begin{array}{c} i\\ j \end{array}\right){\mathrm{LLE}}^{j}\;$$

Finally, LLE can be estimated from the following dependence:

(27) $$\frac{\lambda_{map}}{T}=\;\mathrm{LLE}\;+\;\mathrm{CE}$$

**Figure 10:**
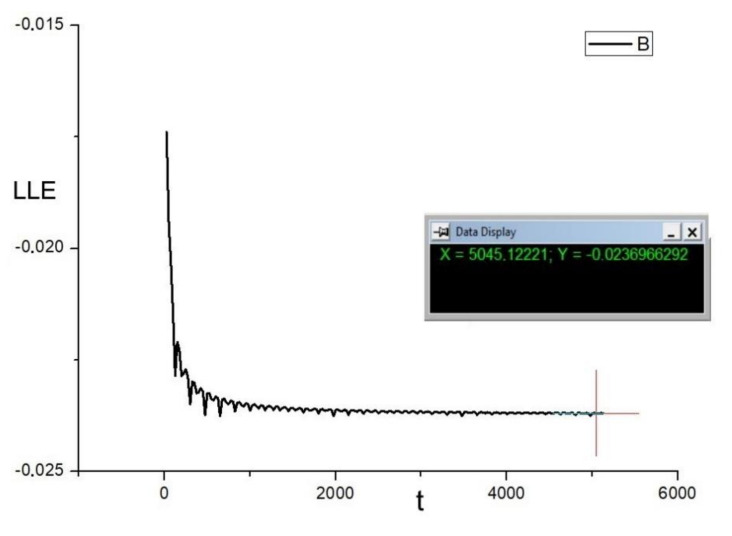
Time series of the largest Lyapunov exponent of the Duffing system. α = 1, β = 0.05, ω = 0.47.

**Figure 11:**
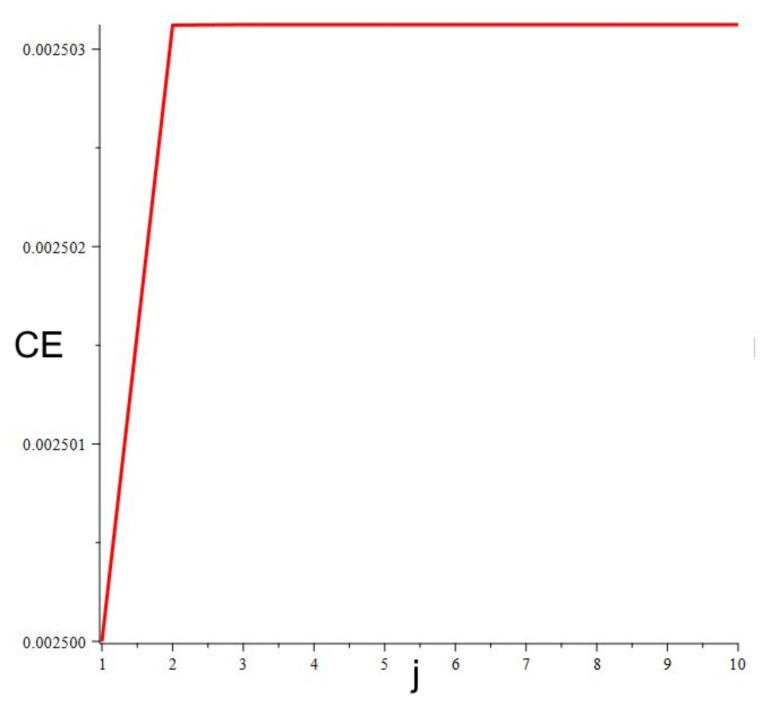
Correction error dependent on number of components (Equation (26)).

The presented approach was applied to estimate LLE of the Duffing system (Equations (16) and (17)). Time series of LLE obtained from numerical simulations can be seen in Figure 10. As is evident, the estimated value of LLE = −0.0237. As the dumping coefficient b = −0.05 and the system remains within the range of the periodic regime LLE = b/2 = −0.025. Thus, the error of the estimated value is 0.0013. From Figure 11, it can be seen that the correction error is within the range of 0.0025. After correction of the obtained LLE value, finally LLE = −0.0262. It means that the error of the presented LLE estimation is about 5%. However, as the value of LLE is computed only while the trajectory intersects the map, the method is expected to be much faster than the continuous ones. In our next article, we will present an extended study of the presented method.

## 5. Conclusions

The present article introduces new methods of LLE estimation for continuous systems and maps. 

We have proved that the sum of dimensionless perturbations, averaged per time unit of measuring the evolution of the system, constitutes the value of the LLE. We have shown that this approach works also in the case of dynamical maps. Additionally, we have proved that LLE can be also equated to the average speed of perturbation change.

The basic background of the methods was presented. The results were compared with other methods. Investigations were carried out for two typical nonlinear systems. We have shown a good agreement of the results obtained with the use of the new approaches with respect to the other methods. 

In the case of continuous systems, we have also compared efficiencies of algorithms based on these methods. We have shown that the new presented methods have better efficiency than the commonly applied M3. We have shown that M1 can save about 7% of the computation time. Method M2 is faster and saves on average about 14% of the computation time. We have also shown that the fastest method, M5, saves on average about 16–22% of the computation time.

We have also discussed basic aspects of the application of the presented methods in estimation of LLE from maps and for noncontinuous systems and showed the initial results of our approach. An extended study of this section of the article will be presented in the next publication.

## Data Availability

The data that support the findings of this study are available from the corresponding author upon reasonable request.
